# Supplementary material for: Revealing the atomic and electronic mechanism of human manganese superoxide dismutase product inhibition
Source: Nat Commun. 2024 Jul 16;15:5973. doi: 10.1038/s41467-024-50260-w (PMC11252399; doi:10.1038/s41467-024-50260-w)
Supplement: Supplementary file 1 — Supplementary Information [file 41467_2024_50260_MOESM1_ESM.pdf]

Supplementary Information

Revealing the atomic and electronic mechanism of human manganese superoxide dismutase product-inhibition

Jahaun Azadmanesh[1], Katelyn Slobodnik[1], Lucas R. Struble[1], William E. Lutz[1], Leighton Coates[2], Kevin L. Weiss[3], Dean A. A. Myles[3], Thomas Kroll[4], and Gloria E. O. Borgstahl[1]*

[1]Eppley Institute for Cancer and Allied Diseases, 986805 Nebraska Medical Center, Omaha, NE 68198-6805, USA

[2]Second Target Station, Oak Ridge National Laboratory, 1 Bethel Valley Road, Oak Ridge, TN 37831, USA

[3]Neutron Scattering Division, Oak Ridge National Laboratory, 1 Bethel Valley Road, Oak Ridge, TN 37831, USA

[4]Stanford Synchrotron Radiation Lightsource, SLAC National Accelerator Laboratory, Menlo Park, CA 94025, USA

*gborgstahl@unmc.edu

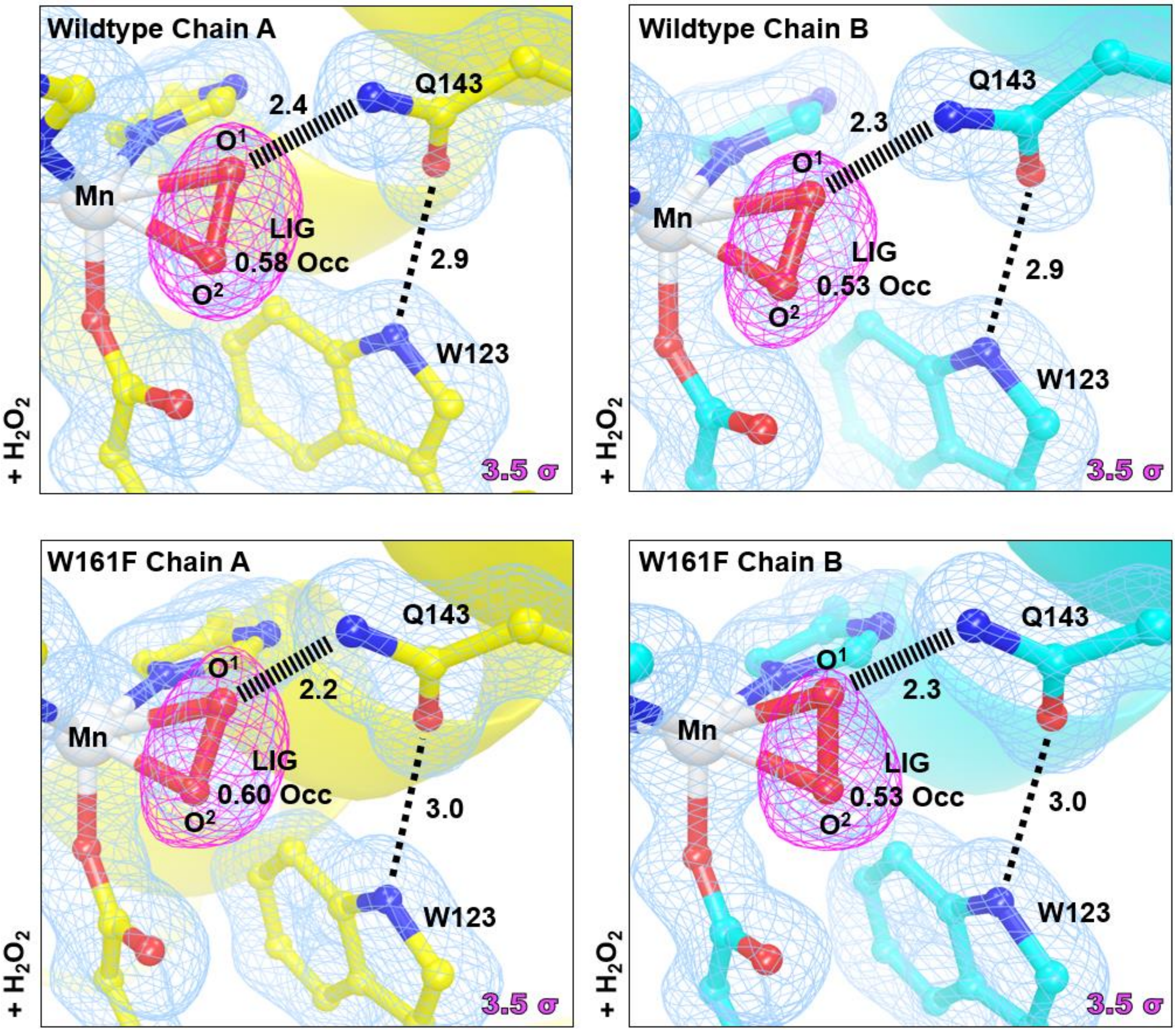

Supplementary Figure 1. X-ray structures of Wildtype and Trp161Phe MnSOD soaked with H_2O_2. Magenta omit $|F_o|$ - $|F_c|$ difference electron density is displayed at 3.5σ. Light blue $2|F_o|$ - $|F_c|$ density is displayed at 1.0σ. Distances are in Å. Dashed lines indicate typical hydrogen bonds, and hashed lines indicate SSHBs that are hydrogen bonds < 2.8 Å from heavy atom to heavy atom. Note that due to photoreduction effects, the dioxygen species are refined at partial occupancy and have side-on binding orientations compared to the neutron structure counterpart that is absent of photoreduction and was refined at full occupancy and has an end-on binding orientation. The H_2O_2-soaked wildtype structure was solved to 1.76 Å resolution while H_2O_2-soaked Trp161Phe was solved to 1.68 Å resolution. Mn-ligand bond distances are found in **Supplementary Table 1.**

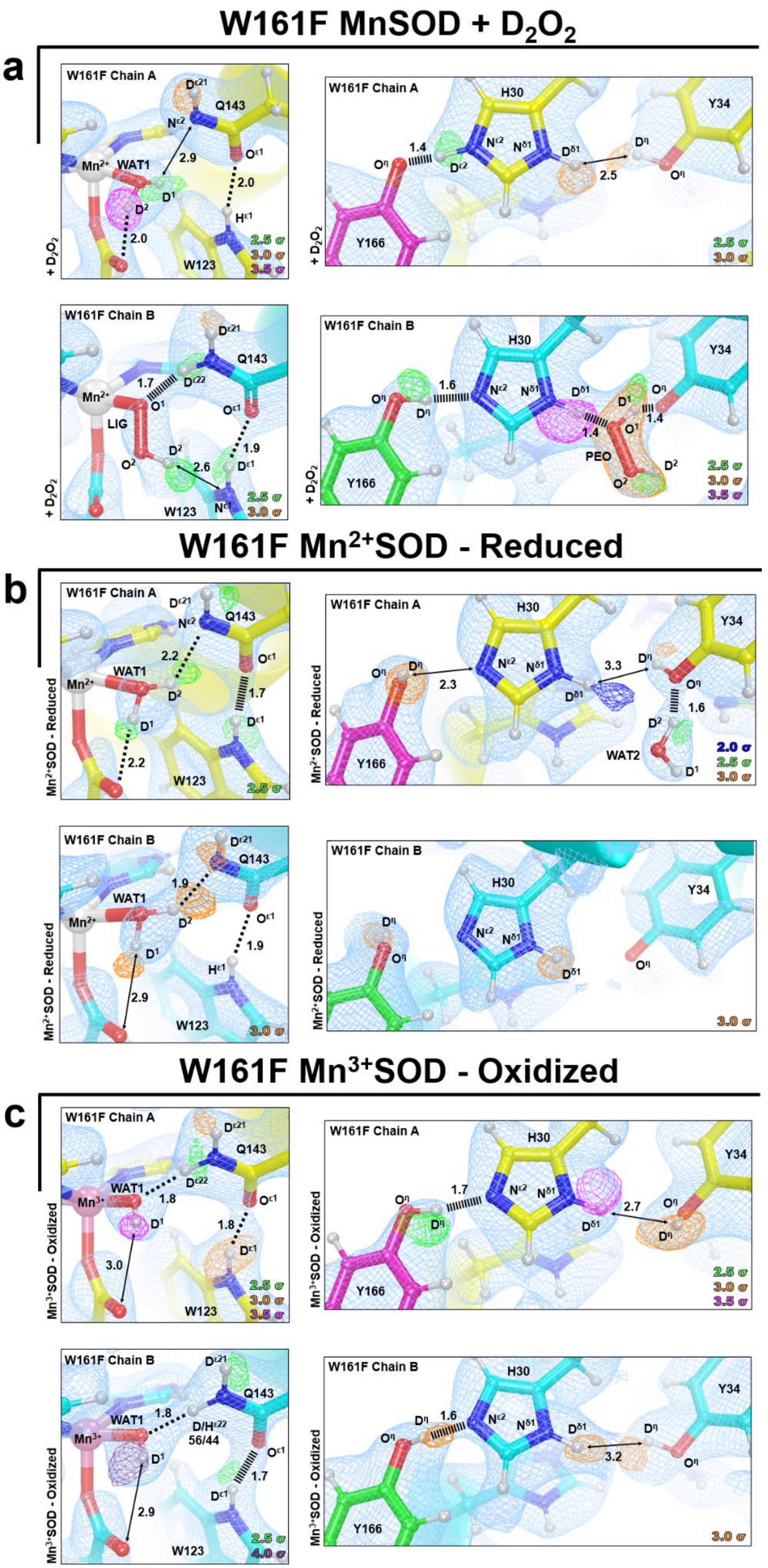

Supplementary Figure 2. Neutron structure and protonation states at the active sites of D_2O_2-soaked, reduced, and oxidized Trp161Phe MnSOD. a Neutron structure of D_2O_2-soaked Trp161Phe MnSOD. **b** Neutron structure of reduced Trp161Phe MnSOD. For $D^{\varepsilon22}$(Q143) in chain B, positive omit difference density was not present above 2.0σ due to density cancellation with the negative neutron scattering length density of hydrogen. As an alternative, the proton position was occupancy refined to yield a ratio of 56% deuterium and 44% hydrogen. Due to a lack of interpretable density for Tyr34 in chain B, a protonation state was not definitively assigned. **c** Neutron structure of oxidized Trp161Phe MnSOD Blue, green, orange, magenta, and purple omit $|F_o|$ - $|F_c|$ difference neutron scattering length density of protons displayed at 2.0 σ, 2.5 σ, 3.0σ, 3.5 σ, and 4.0 σ, respectively. Light blue $2|F_o|$ - $|F_c|$ density is displayed at 1.0σ. Distances are in Å. Dashed lines indicate typical hydrogen bonds, and hashed lines indicate SSHBs, hydrogen bonds < 1.8 Å. All neutron structures were solved to 2.3 Å resolution.

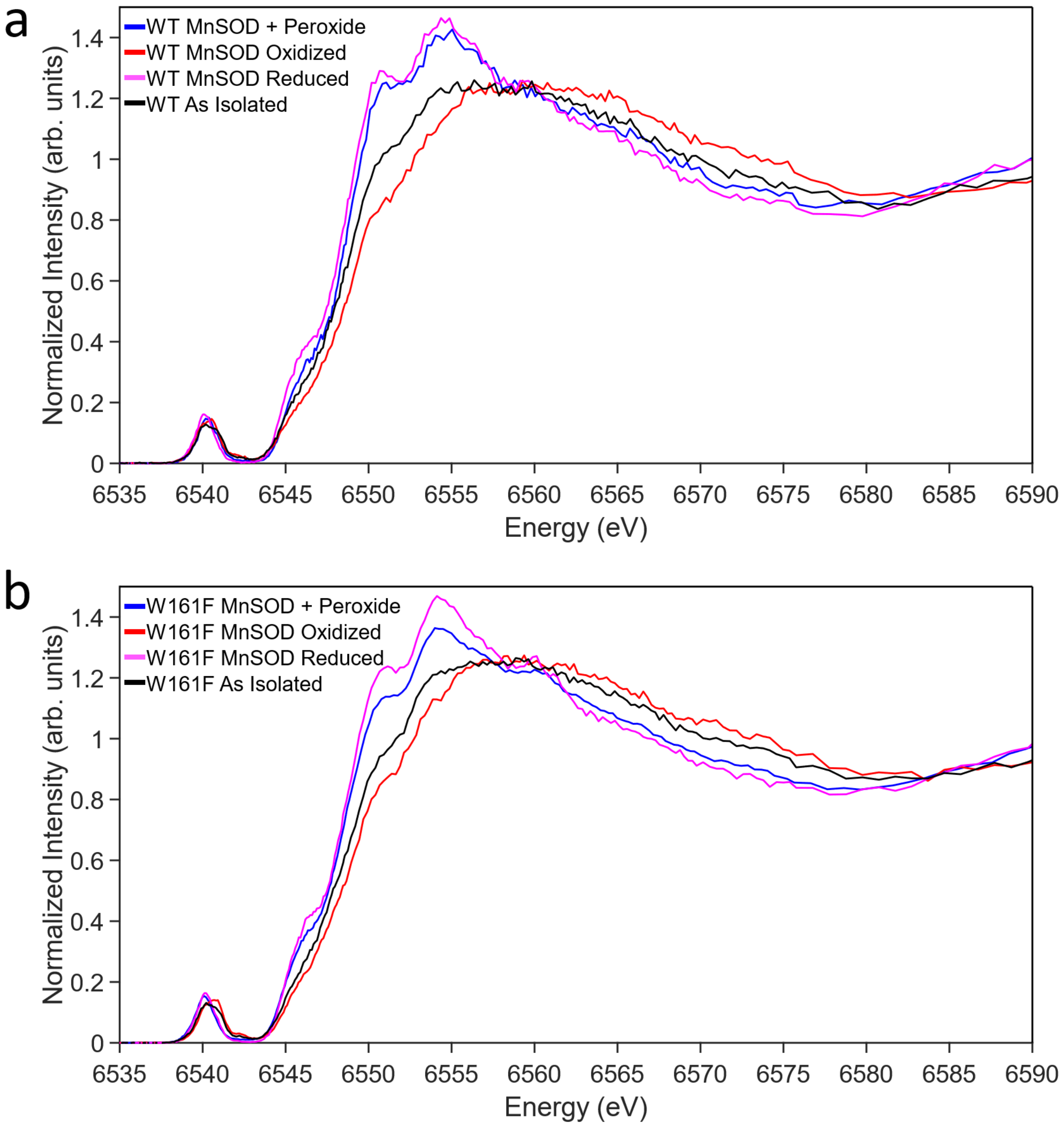

Supplementary Figure 3. Kα HERFD-XANES spectra of MnSOD. **a** HERFD-XANES of wildtype MnSOD in the peroxide-soaked, oxidized, reduced, and as-isolated forms. **b** HERFD-XANES of Trp161Phe MnSOD in the peroxide-soaked, oxidized, reduced, and as-isolated forms. The oxidized and reduced samples correspond to Mn^{3+}SOD and Mn^{2+}SOD resting states. Note that the introduction of peroxide to wildtype MnSOD leads to spectra representing a mixture of dioxygen-bound and reduced complexes. Peroxide mixed with the catalytically deficient Trp161Phe variant leads to distinct spectra representing an isolated dioxygen-bound complex. Data from this figure are provided within the Source Data file.

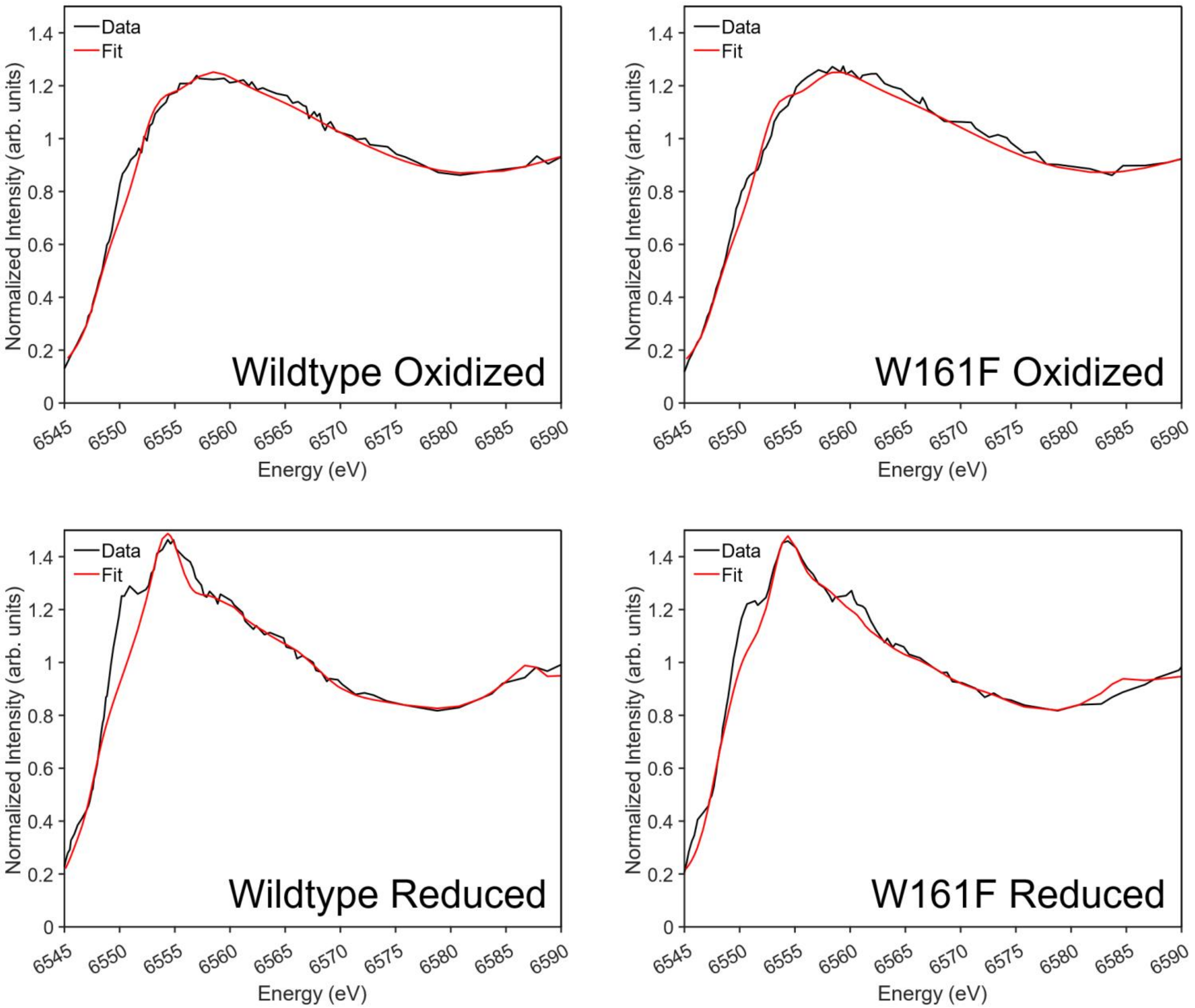

Supplementary Figure 4. Fit of oxidized and reduced HERFD-XANES spectra for wildtype and Trp161Phe MnSOD. Oxidized and reduced samples correspond to Mn^{3+}SOD and Mn^{2+}SOD resting states. Spectra were simulated with FDMNES, and fits were performed using FITIT code. Fits were performed between 6545 and 6590 eV. Data from this figure are provided within the Source Data file.

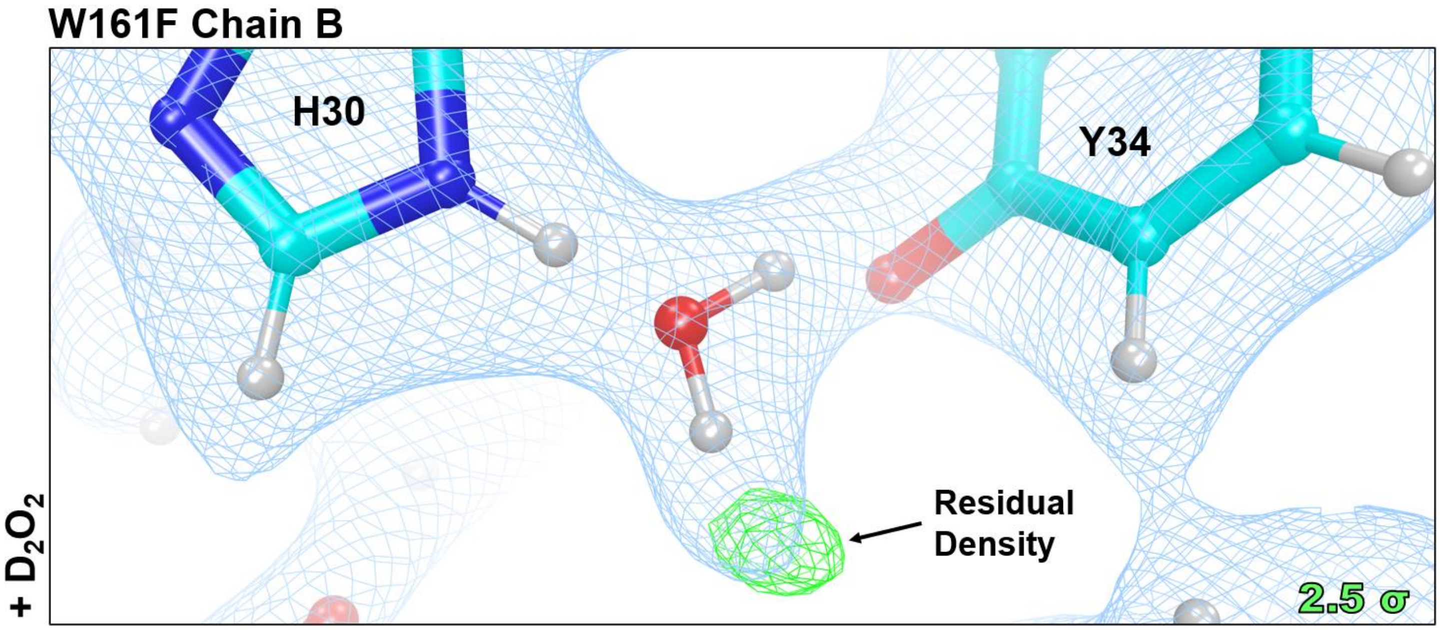

Supplementary Figure 5. Residual density from modelling D_2O in the neutron structure of D_2O_2-soaked Trp161Phe MnSOD. To help verify the identity of the molecule between residues His30 and Tyr34 in Fig. 5a, a D_2O molecule was modeled and refined. The residual $|F_o|$ - $|F_c|$ difference neutron scattering length density indicates the presence of an additional atom and a larger molecule than D_2O. We interpret this residual density to support the presence of a D_2O_2 molecule bound between His30 and Tyr34.

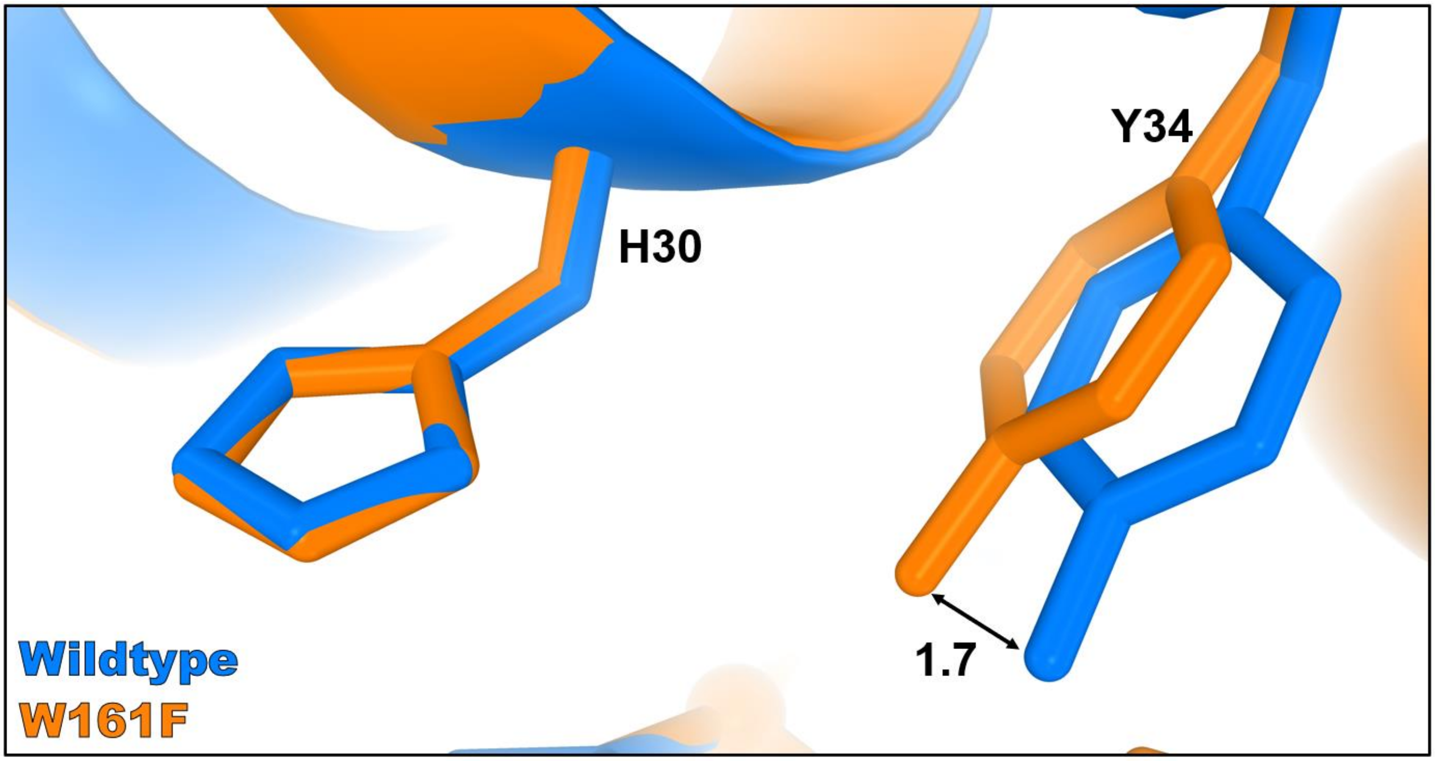

Supplementary Figure 6. Movement of Tyr34 in the Trp161Phe MnSOD variant. Active site overlay of wildtype (blue, PDB ID 5VF9) and Trp161Phe (orange, 8VJ8) MnSOD highlighting a 1.7 Å movement of the Tyr34 hydroxyl group. Superposition was performed by aligning C^{α} atoms of active site residues.

Supplementary Table 1. Active site Mn bond lengths of MnSOD crystal structures.

**Neutron**	Trp161Phe D_2O_2-Soaked[b]		Trp161Phe Mn^{3+}SOD		Trp161Phe Mn^{2+}SOD		Wildtype Mn^{3+}SOD		Wildtype Mn^{2+}SOD[c]	
PDB ID	8VHW		8VJ0		8VHY		7KKS		7KKW	
Mn Bonds (Å)	A	B	A	B	A	B	A	B	A	B
Mn-$N^{\varepsilon 2}$(H26)	2.13	2.20	2.01	2.01	2.20	2.11	2.07	2.07	2.26	2.10
Mn-$N^{\varepsilon 2}$(H74)	2.13	2.28	2.15	2.15	2.19	2.16	2.13	2.12	2.19	2.25
Mn-$O^{\varepsilon 2}$(D159)	2.20	2.05	2.01	2.01	2.13	2.20	1.95	1.94	2.44	2.15
Mn-$N^{\varepsilon 2}$(H163)	2.25	2.17	2.15	2.15	2.14	2.15	2.06	2.14	2.23	2.21
Mn-O(WAT1)	2.13	-	1.84	1.84	2.37	2.24	1.78	1.76	2.12	2.22
Mn-O^1(LIG)[a]	-	1.94	-	-	-	-	-	-	-	-
Mn-O(OL)	-	-	-	-	-	-	-	-	1.82	-
**X-ray**	Trp161Phe H_2O_2-Soaked		Wildtype H_2O_2-Soaked		Trp161Phe Mn^{2+}SOD		Wildtype Mn^{3+}SOD		Wildtype Mn^{2+}SOD	
PDB ID	8VJ4		8VJ5		8VJ8		7KKU		7KLB	
Mn Bonds (Å)	A	B	A	B	A	B	A	B	A	B
Mn-$N^{\varepsilon 2}$(H26)	2.21	2.14	2.16	2.15	2.18	2.19	2.04	2.04	2.18	2.17
Mn-$N^{\varepsilon 2}$(H74)	2.13	2.11	2.18	2.18	2.16	2.17	2.10	2.10	2.28	2.16
Mn-$O^{\varepsilon 2}$(D159)	1.88	2.03	2.07	2.06	2.01	2.03	1.99	1.98	2.09	2.06
Mn-$N^{\varepsilon 2}$(H163)	2.26	2.12	2.21	2.25	2.19	2.18	2.09	2.10	2.25	2.29
Mn-O(WAT1)	-	-	-	-	2.17	2.23	1.92	1.92	2.31	2.28
Mn-O^1(LIG)	2.40	2.36	2.19	2.34	-	-	-	-	-	-
Mn-O^2(LIG)[b]	2.07	2.28	2.20	2.10	-	-	-	-	-	-

[a]O^1(LIG) refers to the closest oxygen atom of the dioxygen species.

[b]Only chain B of D_2O_2-soaked Trp161Phe MnSOD is bound by a dioxygen species, denoted as LIG. Chain A is in the typical five-coordinated state bound by WAT1.

[c]For chain A of wildtype Mn^{2+}SOD, an $^-$OD molecule is observed binding opposite of Asp159 and is six-coordinate. Chain B is in the typical five-coordinated state.

[d]Due to X-ray effects, H_2O_2-soaked X-ray structures, the dioxygen species adopt a side-on conformation of binding and are refined at partial occupancy. See **Supplementary Fig. 1** for the corresponding structures and occupancies.

Supplementary Table 2. EXAFS Fitting Results for H_2O_2-soaked Trp161Phe MnSOD.

**Fit with Amino Acid Ligands and Dioxygen Species (5-coordinate)**														
Mn-O			Mn-N			Mn···C			Mn···O			Mn···O···O		
n	*r* (Å)	σ^2 x 10^3 (Å^2)	*n*	*r* (Å)	σ^2 x 10^3 (Å^2)	*n*	*r* (Å)	σ^2 x 10^3 (Å^2)	*n*	*r* (Å)	σ^2 x 10^3 (Å^2)	*n*	*r* (Å)	σ^2 x 10^3 (Å^2)
2	2.04	2.5	3	2.20	2.5	7	3.15	10	1	2.52	0	2	2.63	0
Mn···C···O			Mn···C···N			χ^2			Reduced χ^2			R-Factor		
n	*r* (Å)	σ^2 x 10^3 (Å^2)	*n*	*r* (Å)	σ^2 x 10^3 (Å^2)	14.28			3.56			0.0197		
2	3.26	0	12	3.48	2.5									
**Fit with Amino Acids Ligands, Dioxygen Species, and Hypothetical Solvent Molecule (6-coordinate)**														
Mn-O			Mn-N			Mn···C			Mn···O			Mn···O···O		
n	*r* (Å)	σ^2 x 10^3 (Å^2)	*n*	*r* (Å)	σ^2 x 10^3 (Å^2)	*n*	*r* (Å)	σ^2 x 10^3 (Å^2)	*n*	*r* (Å)	σ^2 x 10^3 (Å^2)	*n*	*r* (Å)	σ^2 x 10^3 (Å^2)
3	2.05	5.6	3	2.20	5.6	7	3.15	10	1	2.52	0	2	3.16	0
Mn···C···O			Mn···C···N			χ^2			Reduced χ^2			R-Factor		
n	*r* (Å)	σ^2 x 10^3 (Å^2)	*n*	*r* (Å)	σ^2 x 10^3 (Å^2)	29.93			7.47			0.0413		
2	3.25	0	12	3.48	5.6									
**Fit Only with Amino Acid Ligands (4-coordinate)**														
Mn-O			Mn-N			Mn···C			Mn···O			Mn···O···O		
n	*r* (Å)	σ^2 x 10^3 (Å^2)	*n*	*r* (Å)	σ^2 x 10^3 (Å^2)	*n*	*r* (Å)	σ^2 x 10^3 (Å^2)	*n*	*r* (Å)	σ^2 x 10^3 (Å^2)	*n*	*r* (Å)	σ^2 x 10^3 (Å^2)
1	1.99	0	3	2.13	0	7	3.15	10	-	-	-	-	-	-
Mn···C···O			Mn···C···N			χ^2			Reduced χ^2			R-Factor		
n	*r* (Å)	σ^2 x 10^3 (Å^2)	*n*	*r* (Å)	σ^2 x 10^3 (Å^2)	37.08			5.29			0.0511		
2	3.25	0	12	3.48	0									

Supplementary Table 3. Comparison of MnSOD bond lengths from various methods[a].

**Trp161Phe Mn^{3+}SOD**			
Bond	Neutron Structure (Å) Chain A	DFT (Å)	XANES Fit (Å)
Mn-$N^{\varepsilon 2}$(H26)	2.01	2.02	1.96
Mn-$N^{\varepsilon 2}$(H74)	2.15	2.10	2.04
Mn-$N^{\varepsilon 2}$(H163)	2.15	2.08	2.06
Mn-$O^{\delta 2}$(D159)	2.01	1.97	1.96
Mn-O(WAT1)	1.84	1.82	1.80
**Trp161Phe Mn^{2+}SOD**			
Bond	Neutron Structure (Å) Chain A	DFT (Å)	XANES Fit (Å)
Mn-$N^{\varepsilon 2}$(H26)	2.20	2.20	2.14
Mn-$N^{\varepsilon 2}$(H74)	2.19	2.20	2.26
Mn-$N^{\varepsilon 2}$(H163)	2.14	2.19	2.11
Mn-$O^{\delta 2}$(D159)	2.13	2.07	2.08
Mn-O(WAT1)	2.37	2.11	2.37
**Wildtype Mn^{3+}SOD**			
Bond	Neutron Structure (Å) Chain B	DFT (Å)	XANES Fit (Å)
Mn-$N^{\varepsilon 2}$(H26)	2.07	2.06	1.99
Mn-$N^{\varepsilon 2}$(H74)	2.12	2.13	2.08
Mn-$N^{\varepsilon 2}$(H163)	2.14	2.06	2.09
Mn-$O^{\delta 2}$(D159)	1.94	1.95	1.95
Mn-O(WAT1)	1.76	1.80	1.80
**Wildtype Mn^{2+}SOD**			
Bond	Neutron Structure (Å) Chain B	DFT (Å)	XANES Fit (Å)
Mn-$N^{\varepsilon 2}$(H26)	2.10	2.18	2.06
Mn-$N^{\varepsilon 2}$(H74)	2.25	2.24	2.34
Mn-$N^{\varepsilon 2}$(H163)	2.21	2.22	2.25
Mn-$O^{\delta 2}$(D159)	2.15	2.10	2.02
Mn-O(WAT1)	2.22	2.12	2.25

[a]For the neutron structures, only distances of a single chain are shown. Distances for all chains are indicated in Supplementary Table 1.

Supplementary Table 4. Spin populations for Mn-dioxo complexes from DFT.

Structure	*S*	$\rho_s(Mn)$[a]	$\rho_s(O_2)$[a]
$[Mn^{2+}\text{-}^{-}OOH]$	5/2	4.84	0.06
$[Mn^{2+}\text{-}^{\bullet}OOH]$	3	4.86	0.95
$[Mn^{2+}\text{-}^{\bullet}OOH]$[b]	2	4.73	-0.81
$[Mn^{3+}\text{-}^{-}OOH]$	2	4.03	-0.07

[a]The spin density, ρ_s, indicates the net population of unpaired electrons found by spin population analysis. Positive values indicate up-spin electrons while negative values indicate down-spin electrons.

[b]Broken-symmetry DFT was used to acquire negative $\rho_s(O_2)$ from the geometry optimized S=3 coordinates. Geometry optimization with S = 2 collapses to $[Mn^{3+}\text{-}^{-}OOH]$.

Supplementary Table 5. Data collection and refinement statistics for MnSOD.

Data Collection Statistics						
	Neutron			X-ray		
Variant	Trp161Phe	Trp161Phe	Trp161Phe	Trp161Phe	Wildtype	Trp161Phe
Chemical State	D_2O_2-Soaked	Reduced	Oxidized	H_2O_2-Soaked	H_2O_2-Soaked	Reduced
Crystal Growth Environment	Microgravity	Microgravity	Earth	Earth	Earth	Earth
PDB Code	8VHW	8VHY	8VJ0	8VJ4	8VJ5	8VJ8
Diffraction Source	MaNDi			Rigaku FR-E SuperBright		
Temperature (K)	100	100	296	100	100	100
Space group	$P6_122$			$P6_122$		
a, *b*, *c* (Å)	77.80, 77.80, 236.80	78.11, 78.11, 236.32	80.75, 80.75, 239.43	77.66, 77.66, 234.20	78.35, 78.35, 236.63	77.91, 77.91, 235.14
α, β, γ (°)	90, 90, 120			90, 90, 120		
Wavelengths (Å)	2-4			1.5418		
No. of images	12	11	13	252	202	252
Exposure time	48 h	36 h	20 h	300 s	300 s	300 s
No. of unique reflections	19467	18253	20479	48362	38708	46382
Total No. of reflections	189145	76061	122164	256135	235186	456697
Resolution range (Å)	14.82-2.30 (2.38-2.30)	14.74-2.30 (2.42-2.30)	14.89-2.30 (2.42-2.30)	50.00 – 1.68 (1.72-1.68)	50.00 – 1.76 (1.80-1.76)	50.00 – 1.70 (1.74-1.70)
Multiplicity	9.7 (7.0)	4.2 (3.8)	6.0 (5.5)	5.3 (4.1)	6.1 (3.9)	9.8 (5.4)
I/σ(I)	9.7 (5.6)	4.8 (3.1)	5.9 (3.8)	11.41 (2.02)	5.96 (2.24)	16.51 (1.99)
R_{merge}	0.279 (0.286)	0.247 (0.306)	0.202 (0.279)	-	-	-
R_{meas}	0.294 (0.306)	0.278 (0.347)	0.220 (0.307)	0.135 (0.391)	0.289 (0.602)	0.122 (0.579)
$CC_{1/2}$	0.919 (0.607)	0.840 (0.407)	0.978 (0.368)	0.920 (0.769)	0.872 (0.732)	0.950 (0.814)
R_{pim}	0.086 (0.102)	0.122 (0.155)	0.083 (0.125)	0.056 (0.194)	0.104 (0.278)	0.036 (0.244)
Data completeness (%)	99.0 (98.0)	92.6 (87.2)	96.6 (96.4)	99.3 (97.2)	88.4 (78.8)	97.6 (92.5)
Refinement Statistics						
R_{work}	0.2488	0.2752	0.2806	0.1952	0.1993	0.1913
R_{free}	0.2774	0.3005	0.3089	0.2422	0.2183	0.2237
[a]No. of atoms<br>Protein<br>[b]Solvent<br>Mn	 6385<br>725<br>2	 6540<br>1392<br>2	 6228<br>479<br>2	 3168<br>460<br>2	 3174<br>614<br>2	 3168<br>570<br>2
R.m.s. deviations<br>Bond lengths (Å)<br>Bond angles (°)	 0.003<br>0.58	 0.002<br>0.53	 0.003<br>0.53	 0.011<br>1.05	 0.003<br>0.58	 0.004<br>0.69
Average *B*-factor<br>Protein<br>Water<br>Mn<br>Peroxide	20.71<br>21.13<br>17.40<br>19.40<br>21.58	11.67<br>11.44<br>13.25<br>7.12<br>-	17.42<br>17.62<br>13.76<br>9.37<br>-	19.34<br>18.59<br>24.55<br>14.11<br>13.22	21.90<br>20.15<br>30.85<br>14.47<br>14.63	16.48<br>15.20<br>23.26<br>10.93<br>-
[c]Coordinate Error (Å)	0.28	0.33	0.36	0.23	0.18	0.23

[a]No. of atoms includes H/D atoms for neutron structures.

[b]The neutron structure of oxidized Trp161Phe was collected at room temperature and, as a result, has fewer solvent atoms compared to the other structures.

[c]Estimated coordinate error determined by PHENIX. Note that the disorder of atom positions is reflected by the *B*-factor.
