# Supplementary material for: Revealing the atomic and electronic mechanism of human manganese superoxide dismutase product inhibition
Source: Nat Commun. 2024 Jul 16;15:5973. doi: 10.1038/s41467-024-50260-w (PMC11252399; doi:10.1038/s41467-024-50260-w)
Supplement: Supplementary file 3 — Reporting Summary [file 41467_2024_50260_MOESM3_ESM.pdf]

nature portfolio

Corresponding author(s): Gloria Borgstahl

Last updated by author(s): Jul 4, 2024

Reporting Summary

Nature Portfolio wishes to improve the reproducibility of the work that we publish. This form provides structure for consistency and transparency in reporting. For further information on Nature Portfolio policies, see our Editorial Policies and the Editorial Policy Checklist.

Statistics

For all statistical analyses, confirm that the following items are present in the figure legend, table legend, main text, or Methods section.

n/a	Confirmed	
☒	☐	The exact sample size (*n*) for each experimental group/condition, given as a discrete number and unit of measurement
☐	☒	A statement on whether measurements were taken from distinct samples or whether the same sample was measured repeatedly
☒	☐	The statistical test(s) used AND whether they are one- or two-sided<br>*Only common tests should be described solely by name; describe more complex techniques in the Methods section.*
☒	☐	A description of all covariates tested
☐	☒	A description of any assumptions or corrections, such as tests of normality and adjustment for multiple comparisons
☒	☐	A full description of the statistical parameters including central tendency (e.g. means) or other basic estimates (e.g. regression coefficient) AND variation (e.g. standard deviation) or associated estimates of uncertainty (e.g. confidence intervals)
☒	☐	For null hypothesis testing, the test statistic (e.g. *F*, *t*, *r*) with confidence intervals, effect sizes, degrees of freedom and *P* value noted<br>*Give P values as exact values whenever suitable.*
☒	☐	For Bayesian analysis, information on the choice of priors and Markov chain Monte Carlo settings
☒	☐	For hierarchical and complex designs, identification of the appropriate level for tests and full reporting of outcomes
☒	☐	Estimates of effect sizes (e.g. Cohen's *d*, Pearson's *r*), indicating how they were calculated

Our web collection on statistics for biologists contains articles on many of the points above.

Software and code

Policy information about availability of computer code

Data collection	MaNDi, HKL-3000 v717, SPEC v6, WebXAS v2023
Data analysis	Mantid v6.0.0. Lauenorm v6.0, HKL-3000 v717, Phenix v1.21, Coot v0.9.6, Larch v0.9.78, Matlab v2023a, Orca v5.0, FEFF v8L, FDMNES v2023, Fitit v2021

For manuscripts utilizing custom algorithms or software that are central to the research but not yet described in published literature, software must be made available to editors and reviewers. We strongly encourage code deposition in a community repository (e.g. GitHub). See the Nature Portfolio guidelines for submitting code & software for further information.

Data

Policy information about availability of data

All manuscripts must include a data availability statement. This statement should provide the following information, where applicable:

- Accession codes, unique identifiers, or web links for publicly available datasets
- A description of any restrictions on data availability
- For clinical datasets or third party data, please ensure that the statement adheres to our policy

Coordinates and structure factors for neutron and X-ray crystallographic data generated in this study have been deposited in the Protein Data Bank under the following accession codes (8VHW [https://doi.org/10.2210/pdb8vhw/pdb], 8VHY [https://doi.org/10.2210/pdb8vhy/pdb], 8VJ0 [https://doi.org/10.2210/pdb8vj0/pdb], 8VJ4 [https://doi.org/10.2210/pdb8vj4/pdb], 8VJ5 [https://doi.org/10.2210/pdb8vj5/pdb], and 8VJ8 [https://doi.org/10.2210/pdb8vj8/pdb]). Previously published wildtype neutron structures are available in the Protein Data Bank under the following accession codes (7KKS [https://doi.org/10.2210/pdb7KKS/pdb] and

7KKW [https://doi.org/10.2210/pdb7KKW/pdb]). X-ray spectroscopy data and coordinates for computational models are provided in the Source Data file. All relevant data supporting the key findings of this study are available within this article, its Supplementary Information, or in the Source data file. Additional raw data is available from the corresponding author upon request.

Research involving human participants, their data, or biological material

Policy information about studies with human participants or human data. See also policy information about sex, gender (identity/presentation), and sexual orientation and race, ethnicity and racism.

Reporting on sex and gender	Human subjects were not used.
Reporting on race, ethnicity, or other socially relevant groupings	Human subjects were not used.
Population characteristics	Human subjects were not used.
Recruitment	Human subjects were not used.
Ethics oversight	Human subjects were not used.

Note that full information on the approval of the study protocol must also be provided in the manuscript.

Field-specific reporting

Please select the one below that is the best fit for your research. If you are not sure, read the appropriate sections before making your selection.

☐ Life sciences ☐ Behavioural & social sciences ☐ Ecological, evolutionary & environmental sciences

For a reference copy of the document with all sections, see nature.com/documents/nr-reporting-summary-flat.pdf

Life sciences study design

All studies must disclose on these points even when the disclosure is negative.

Sample size	Data were collected from single samples (crystals, solution samples).
Data exclusions	Data with low signal were excluded.
Replication	Data were collected from single samples (crystals, solution samples).
Randomization	Data were collected from single samples (crystals, solution samples).
Blinding	No blinding was used in this study.

Behavioural & social sciences study design

All studies must disclose on these points even when the disclosure is negative.

Study description	This was not a behavioral/social study.
Research sample	This was not a behavioral/social study.
Sampling strategy	This was not a behavioral/social study.
Data collection	This was not a behavioral/social study.
Timing	This was not a behavioral/social study.
Data exclusions	This was not a behavioral/social study.
Non-participation	This was not a behavioral/social study.
Randomization	This was not a behavioral/social study.

Ecological, evolutionary & environmental sciences study design

All studies must disclose on these points even when the disclosure is negative.

Study description	This was not a ecological, evolutionary, or environmental study.
Research sample	This was not a ecological, evolutionary, or environmental study.
Sampling strategy	This was not a ecological, evolutionary, or environmental study.
Data collection	This was not a ecological, evolutionary, or environmental study.
Timing and spatial scale	This was not a ecological, evolutionary, or environmental study.
Data exclusions	This was not a ecological, evolutionary, or environmental study.
Reproducibility	This was not a ecological, evolutionary, or environmental study.
Randomization	This was not a ecological, evolutionary, or environmental study.
Blinding	This was not a ecological, evolutionary, or environmental study.

Did the study involve field work? ☐ Yes ☒ No

Reporting for specific materials, systems and methods

We require information from authors about some types of materials, experimental systems and methods used in many studies. Here, indicate whether each material, system or method listed is relevant to your study. If you are not sure if a list item applies to your research, read the appropriate section before selecting a response.

Materials & experimental systems

n/a	Involved in the study
☒	☐ Antibodies
☒	☐ Eukaryotic cell lines
☒	☐ Palaeontology and archaeology
☒	☐ Animals and other organisms
☒	☐ Clinical data
☒	☐ Dual use research of concern
☒	☐ Plants

Methods

n/a	Involved in the study
☒	☐ ChIP-seq
☒	☐ Flow cytometry
☒	☐ MRI-based neuroimaging

Plants

Seed stocks	Plants were not used in this study.
Novel plant genotypes	Plants were not used in this study.
Authentication	Plants were not used in this study.
